# Type of Exercise and Factors Contributing to Exercise Adherence Among Diabetes Patients in Saudi Arabia

**DOI:** 10.7759/cureus.86146

**Published:** 2025-06-16

**Authors:** Tameem A Alhomaid, Khalid A Alkhalifah, Abdullah S Aljudayi, Omar A Alharbi, Asim A Aladwan, Fahad A Alresheedi

**Affiliations:** 1 Family Medicine, Qassim Health Cluster, Buraydah, SAU; 2 Medicine, Qassim University, Buraydah, SAU

**Keywords:** diabetes mellitus, exercise adherence, patient adherence, physical exercise, saudi arabia

## Abstract

Aim and background: Diabetes mellitus (DM) management guidelines are continuously updated to reflect emerging evidence and changes in patient lifestyles. Physical activity is considered essential to achieving adequate blood glucose control. The study aims to evaluate the level of exercise adherence among diabetic patients in Saudi Arabia and the factors that affect adherence.

Method: This was an observational cross-sectional study; the data collection tool included a questionnaire distributed across healthcare centers in Saudi Arabia. The questionnaire included a socio-demographic section, medical history of diabetes, exercise knowledge and awareness, and factors affecting exercise adherence. Participants included were 18 years or older, diagnosed with type 1 or type 2 diabetes, and had documented HbA1c levels. IBM SPSS Statistics for Windows, version 24 (Released 2016; IBM Corp., Armonk, New York, United States) was used for data analysis. The result was considered statistically significant if p-value<0.05.

Results: Out of 1400 participants, 60.2% (n=843) were female and 39.8% (n=557) were male. (31%) reported engaging in regular exercise more than 150 minutes per week. Walking (45%), resistance training (30%), and cycling (25%) were the most common activities. Exercise-adherent individuals had better glycemic control levels compared to non-adherents (P-value 0.001). Empowering factors were social support and access to resources, while time constraints and health limitations were major barriers to achieving adherence.

Conclusion: The study shows a low level of adherence among participants. Walking is the most preferred exercise. The younger population was more adherent to exercise. The study underscores the importance of addressing barriers and enhancing support systems to improve exercise adherence among diabetic patients in Saudi Arabia.

## Introduction

Diabetes mellitus (DM) has become a critical global health issue, set apart by its rising prevalence and its significant effect on personal quality of life. The World Health Organization (WHO) estimates that 830 million individuals experience the ill effects of diabetes, a condition described by ongoing hyperglycemia because of insulin resistance or deficiency in insulin secretion [1]. The increased prevalence of DM is a main pressing issue, especially in Saudi Arabia, where it ranks seventh worldwide and second in the Middle East [2]. Currently, it is estimated that seven million people in Saudi Arabia are diagnosed with DM, with an additional three million labeled as prediabetes, mirroring a basic requirement for viable management methodologies [2].

Effective management of diabetes is multifaceted, including pharmacological management combined with lifestyle modification [3]. Among lifestyle changes, physical exercise plays a crucial part that offers various advantages, including improved glycemic control and insulin responsiveness, and diminished risk of cardiovascular comorbidities [4]. The American Diabetes Association (ADA) suggests that people with diabetes participate in 150 minutes of moderate-intensity or vigorous exercise each week, combined with resistance training on at least two days every week [5]. This helps in controlling blood glucose levels and weight, and leads to general well-being.

The significance of exercise in diabetes management is upheld by multiple studies. For example, resistance training has been shown to improve glycemic control by increasing muscle mass and improving insulin responsiveness. Studies have shown that resistance exercise can prompt a critical reduction in glycated hemoglobin (HbA1c) levels, a vital marker of long-term glucose control [6]. Then again, aerobic exercises, like walking or cycling, are valuable for cardiovascular wellbeing and weight management, contributing to better diabetes management [7]. Nonetheless, different examinations recommend that both aerobic and resistance activities can be similarly compelling in managing diabetes [8,9].

Physical exercise has been shown to improve the general psychological wellness of diabetes patients. It decreases feelings of anxiety and further improves mental health [10,11]. Exercise improves quality of life and decreases the risk of comorbidities related to diabetes, like cardiovascular disease and neuropathy [12]. Enhanced quality of life upholds better adherence to diabetes management plans and leads to a more active and satisfying lifestyle.

Notwithstanding the irrefutable advantages of exercise, adherence to physical activity among diabetes patients remains a significant challenge. Research shows that adherence rates are strikingly low, especially in the Gulf Countries, including Saudi Arabia. For instance, a study conducted in Kuwait uncovered that 64.4% of participants didn't participate in any type of exercise, specifying lack of time and ailments as significant hindrances [13]. Multiple studies indicate that 30-35.8% of people living with diabetes have integrated exercise into their diabetes management schedules [14,15]. Factors contributing to these low adherence rates include a lack of family support, social hindrances, and natural constraints, like restricted access to safe exercise facilities [14,15].

Understanding the factors that impact exercise adherence is critical for creating suitable exercise types and variations. Empowering factors like social help, support, and access to resources can improve adherence, while deterring factors like environmental issues, lack of time, and physical disabilities can hinder adherence to physical activity. Recognizing these factors and understanding their effect on exercise adherence will give significant bits of knowledge into how to design and carry out techniques that advance active work among people living with diabetes [6,16].

This study aims to highlight these issues by estimating the degree of exercise adherence among diabetes patients in Saudi Arabia, recognizing the favored types of exercise, and investigating the factors that increase or decrease adherence. By gaining a complete comprehension of these components, the study intends to develop personalized strategies to improve exercise adherence that eventually enhance diabetes management results in the locale.

Rationale

Multiple researchers have proven the benefit of exercise as part of diabetic management [6,7,10-12]. However, the level of adherence to physical activity is considerably low in Saudi Arabia. Different barriers, such as environmental, cultural, and social factors, could contribute to this gap. In this study, we measured the level of adherence among the Saudi population to see if there is any change, in addition to identifying the preferred type of exercise and factors affecting their adherence.

Research objectives

The essential target of this study is to quantify the degree of exercise adherence among diabetes patients in Saudi Arabia. Additional goals include deciding the favored types of exercise among diabetes patients, distinguishing the factors that support or hinder exercise adherence, and recognizing the type of activity and factors contributing to exercise adherence among diabetes patients in Saudi Arabia.

## Materials and methods

This was an observational, cross-sectional study conducted in multiple primary healthcare centers from different health clusters across Saudi Arabia over a period of six months. The study was approved by the Regional Research Ethics Committee, General Directorate of Health Affairs, Al-Qassim Province (approval number: 607/45/14991). Data were anonymized to guarantee the privacy of respondents.

Eligibility

The inclusion criteria were patients from Saudi Arabia aged 18 years or above diagnosed with type 1 DM (T1DM) or type 2 DM (T2DM) for at least one year with well-documented glycated hemoglobin (HbA1C) levels. Participants younger than 18 years old, diagnosed with DM for less than one year, or missing information on HbA1c level were excluded from the study. 

Data collection tool

For data collection, a questionnaire (see Appendices) was designed based on previous literature and feedback from two family medicine consultants in the Qassim region. It had five sections; the first section included demographic features, the second section included information on the current medical history of diabetes, the third section included assessment of diabetes care and exercise knowledge among participants, the fourth section included exercise adherence level assessment, and the fifth section included supporting and constraining factors affecting their adherence. 

The questionnaire was administered across the kingdom with the help of data collectors who are not co-authors. The questionnaires were distributed among Health Center visitors at Chronic Disease Clinics.

Sample size calculation

The head of the Middle East Pharmaceutical Vigilance Organization and a member of the Pharmaceutical Errors Committee at the WHO, Dr. Thamer Al-Shammari, estimated the number of people with diabetes in Saudi Arabia at around 3.7 million. It costs the national economy approximately 15% of the total health budget in Saudi Arabia [17]. With a population of 3700000 [17], a confidence level of 95%, a margin of error of 5%, and the population proportion of 50%, the sample size calculated was 385 to account for possible variations and guarantee adequate ability to recognize significant differences in exercise adherence and its impacting factors. A pilot study with 100 participants was conducted to refine the questionnaire and test its reliability and validity.

Data management and analysis plan 

The collected data were analyzed by the Statistical Package for the Social Sciences (SPSS) version 24. The analysis incorporated descriptive statistics, relationship examinations, and other tests to investigate exercise adherence and influencing factors. Participants who did physical exercise for 150 minutes of moderate-power high-impact exercise each week were considered exercise adherents. Chi-square test was used to assess the association between exercise adherence and different variables. Analyzing the factors that affect exercise adherence, we designed a scale from 1 (not important) to 5 (vital). p-value <0.05 was considered statistically significant.

## Results

The study included 1400 diabetic patients across Saudi Arabia. As shown in Table 1, the analysis revealed that 60.2% of participants were female and 39.8% were male. In terms of age, 37.8% were younger than 40 years. Among participants aged 40 years or older, 25.1% were between 40-49 years, 22.1% between 50-59 years, 12.1% between 60-69 years, and 2.9% were aged 70 years or older. Regarding nationality, 94.6% were Saudi nationals and most participants were married (67.1%). Educational attainment varied, with 41.6% holding a university degree, 21.5% having completed high school, 15.7% holding a diploma, 8.6% having primary education, and 5.4% having intermediate-level education. A minority (7.1%) were illiterate. Regionally, participants were predominantly from the Central (37.5%), Western (24.7%), and Eastern (24.4%) regions. Smaller proportions were from the Southern (10.2%) and Northern (3.2%) regions.

**Table 1:** Demographic features of participants (N= 1400)

Variable	Category	Frequency	Percentage
Gender	Females	843	60.2%
	Male	557	39.8%
Age (years)	<40	529	37.8%
	40-49	352	25.1%
	50-59	309	22.1%
	60-69	170	12.1%
	70+	40	2.9%
Nationality	Saudi	1324	94.6%
	Non-Saudi	76	5.4%
Marital status	Married	940	67.1%
	Single	276	19.7%
	Widowed	119	8.5%
	Divorced	65	4.6%
Academic Qualification	Illiterate	99	7.1%
	Primary	121	8.6%
	Intermediate	76	5.4%
	High School	301	21.5%
	Diploma	220	15.7%
	University	583	41.6%
Region	Central Region	525	37.5%
	Western Region	346	24.7%
	Eastern Region	341	24.4%
	Southern Region	143	10.2%
	Northern Region	45	3.2%

Regarding the medical profile of our participants, the data showed that the majority of individuals in the study had T2DM, representing 85.6% of the sample, while only 14.4% had T1DM. Regarding the duration of diabetes, 37.5% of participants had been diagnosed for one to five years, 33.4% for 5-10 years, and 29.1% for more than 10 years. In terms of treatment type, 41.6% of participants were managing their diabetes using oral hypoglycemic agents (OHA), 24.4% were using a combination of OHA and insulin, 21.8% relied on lifestyle modifications, and 12.2% were on insulin injections alone. In terms of glycemic control, 28.2% of participants had HbA1C levels below 7%, indicating good control. Additionally, 37.8% had HbA1C levels between 7.0% and 8.4%, 22.4% fell in the 8.5-9.9% range, and 11.6% had HbA1C levels of 10% or higher (Table 2).

**Table 2:** Medical profile of participants (N=1400) HbA1C: glycated hemoglobin; OHA: oral hypoglycemic agent

Variable	Category	Frequency	Percentage
Type of Diabetes	Type 1	202	14.4%
	Type 2	1198	85.6%
Duration of diabetes	1-5 years	525	37.5%
	5-10 years	468	33.4%
	More than 10 years	407	29.1%
Type of treatment	Lifestyle modification	305	21.8%
	OHA	583	41.6%
	Insulin injection	171	12.2%
	Mixed OHA and insulin	341	24.4%
HbA1C	Less than 7%	395	28.2%
	7-8.4%	529	37.8%
	8.5-9.9%	314	22.4%
	10% and above	162	11.6%

**Figure 1:**
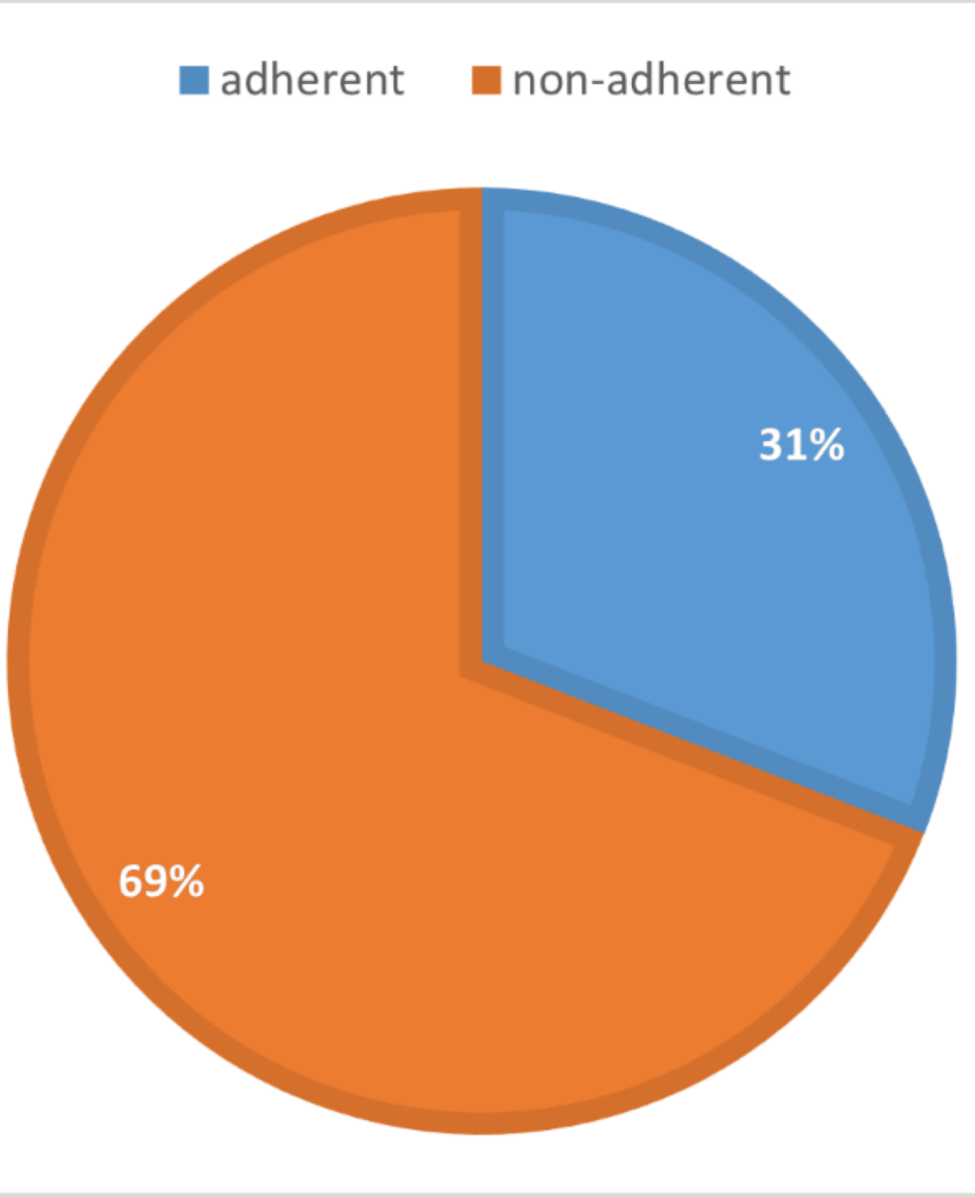
Exercise adherence level among participants (N=1400)

**Figure 2:**
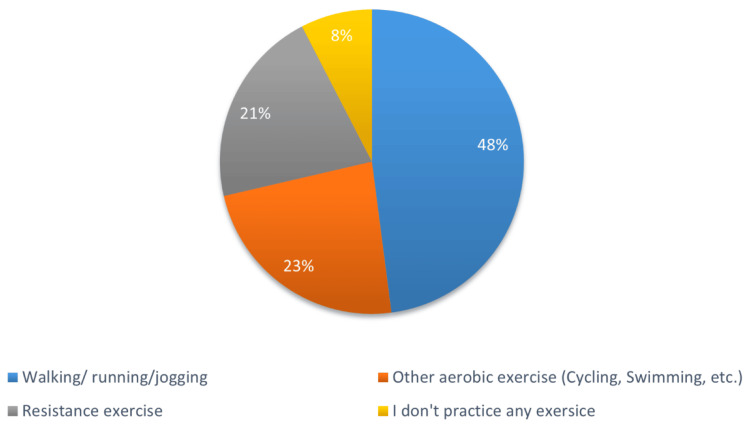
Favored type of exercise among participants (N=1400)

Figure 1 and Figure 2 depict exercise adherence level and favored type of exercise, respectively, among the participants. Out of the total respondents, only 31% (n=433) did physical exercise for 150 minutes of moderate-power high-impact exercise each week and could be considered exercise adherents. The remaining 69% (n=967) were sorted as non-adherent (Figure 1). Regarding preferred types of physical activity, 48% (n=671) of responses stated that walking was their favored exercise, 23% (n=328) practiced other types of aerobic exercise, and 21% (n=294) practiced resistance training; 8% (n=107) of respondents declared that they did not perform any type of exercise (Figure 2).

Table 3 analyzes the relationship between exercise adherence and demographic features. Gender, age, and education level show statistically significant results with p-values of 0.005, 0.001, and 0.001, respectively. Only regions show statistically insignificant results with a p-value of 0.198. Based on gender, male participants are more adherent when compared with female participants (35.2% vs 28.1%). Regarding age, participants aged less than 50 years showed a higher adherence rate compared with older groups. The lowest adherence was among the 50-59 age group (15.5%). Participants with a bachelor’s degree or higher had the highest adherence (38.6%). Adherence was lowest among participants with intermediate education (11.8%). Despite statistically insignificant results between exercise adherence and region (p-value 0.198), the Southern (34.3%) and Central (33.7%) regions of Saudi Arabia showed a higher adherence rate than the other regions. The Northern region had the lowest adherence (22.2%). 

**Table 3:** Relationship between exercise adherence and demographic features Statistical test: Chi-square. P-value <0.05 is statistically significant.

Variable	Characteristics	Not adherent, n (%)	Adherent, n (%)	P-value
Gender	Male	361 (64.8%)	196 (35.2%)	0.005
	Female	606 (71.9%)	237 (28.1%)	
Age	< 40 years	326 (61.6%)	203 (38.4%)	0.001
	40–49 years	219 (62.2%)	133 (37.8%)	
	50–59 years	261 (84.5%)	48 (15.5%)	
	60–69 years	135 (79.4%)	35 (20.6%)	
	≥ 70 years	26 (65.0%)	14 (35.0%)	
Academic Qualification	Illiterate	76 (76.8%)	23 (23.2%)	0.001
	Primary	92 (76.0%)	29 (24.0%)	
	Intermediate	67 (88.2%)	9 (11.8%)	
	High School	212 (70.4%)	89 (29.6%)	
	Diploma	162 (73.6%)	58 (26.4%)	
	Bachelor	358 (61.4%)	225 (38.6%)	
Region	Central	348 (66.3%)	177 (33.7%)	0.198
	Western	249 (72.0%)	97 (28.0%)	
	Eastern	241 (70.7%)	100 (29.3%)	
	Southern	94 (65.7%)	49 (34.3%)	
	Northern	35 (77.8%)	10 (22.2%)	

Table 4 shows the relationship between exercise adherence and diabetes profile. The table shows that T1DM patients (31.7%) were slightly similar in adherence to T2DM patients (30.8%). This difference is not statistically significant (p-value 0.802). Duration of diabetes, type of management, and HbA1c level have statistically significant results (P-value 0.032, 0.001, and 0.00,1 respectively). It shows that patients diagnosed with DM for less than five years, patients on insulin or lifestyle modification, and HbA1c between (7-8.5%) are more adherent than others. On the other hand, those who are diagnosed with DM for more than 10 years, on mixed oral hypoglycemic medication and insulin, and HbA1c more than 10% have a lower level of adherence.

**Table 4:** Relationship between exercise adherence and diabetes profile Statistical test: Chi-square; P-value <0.05 is statistically significant T1DM: type 1 diabetes mellitus; T2DM: tyoe 2 diabetes mellitus

Variable	Characteristics	Not adherent, n (%)	Adherent, n (%)	P-value
Type of diabetes	T1DM	138 (68.3%)	64 (31.7%)	0.802
	T2DM	829 (69.2%)	369 (30.8%)	
Duration of Diabetes	< 5 years	345 (65.7%)	180 (34.3%)	0.032
	5–10 years	322 (68.8%)	146 (31.2%)	
	> 10 years	300 (73.7%)	107 (26.3%)	
Type of Treatment	Life style	197 (64.6%)	108 (35.4%)	0.001
	Oral Hypoglycemic Agent (OHA)	397 (68.1%)	186 (31.9%)	
	Insulin	107 (62.6%)	64 (37.4%)	
	Mixed	266 (78.0%)	75 (22.0%)	
A1C Level	< 7%	278 (70.4%)	117 (29.6%)	0.001
	7–8.5%	330 (62.4%)	199 (37.6%)	
	8.5–10	215 (68.5%)	99 (31.5%)	
	> 10%	144 (88.9%)	18 (11.1%)	

Table 5 illustrates the factors impacting exercise adherence. Respondents were asked to rate different factors that affected their exercise adherence on a scale from 1 (not important) to 5 (vital). The most frequent supporting factors included social help (Mean = 4.2, SD = 0.8) and access to resources (Mean = 3.8, SD = 0.9). The most frequent deterring factors were lack of time (Mean = 4.1, SD = 0.7) and health limitation (Mean = 3.9, SD = 0.8).

**Table 5:** Factors impacting exercise adherence among participants. (1) is least important, (5) is most important

Factor	Mean	Standard Deviation
Social Support	4.2	0.8
Access to Resources	3.8	0.9
Lack of Time	4.1	0.7
Health Limitation	3.9	0.8

## Discussion

The aim of the study was to assess the level of adherence among diabetic patients in Saudi Arabia and investigate the preferred type of exercise and the factors affecting their long-term adherence. The findings offer valuable information about the pattern of physical exercise that encourages exercise adherence. 

Exercise type preferences and popularity

Walking was a commonly preferred exercise among participants. Consistent with global trends, walking emerged as the most prevalent form of exercise, likely due to its low cost, convenience, and adaptability across fitness levels [18]. Resistance training was less common, possibly due to perceived complexity, equipment needs, or social intimidation [19].

Intrinsic and extrinsic motivation

Participants reported both intrinsic (enjoyment, stress relief, and setting a goal to achieve) and extrinsic (weight control, glucose control, good weather, and social approval) motivators. This is consistent with the Self-Determination Theory (SDT), when intrinsic motivation achieves longer adherence when combined with extrinsic rewards [20]. This mirrors trends in digital fitness apps, where initial novelty wears off without deeper engagement.

Supporting factors

Social Support

Social support is a huge factor in exercise adherence [21]. Members who got support and backing from family, companions, or exercise partners reported a higher adherence rate [22]. This finding is consistent with a study from Japan suggesting that social cohesion may facilitate the transition from extrinsic to intrinsic motivation [23]. This aligns with recent work on community-based interventions, which proposes that having a supportive community can improve inspiration and assist people with maintaining their exercise schedules.

Access to Resources

The availability of resources, for example, exercise facilities and community-based programs, impacted adherence (Mean = 3.8, SD = 0.9). Access to these appropriate facilities and programs can give the vital foundation to maintain regular exercise activity [24].

Barriers to exercise

Lack of Time

Absence of time was the most common hindrance to exercise adherence (Mean = 4.1, SD = 0.7). This demonstrates a requirement for adaptable exercise choices that can be squeezed into different lifestyles [25].

Physical Restrictions

Physical restrictions likewise is also one of the most deterring factors in adherence to exercise (Mean = 3.9, SD = 0.8). Conditions like pain, weakness, or complications related to diabetes affect adherence to exercise. Tailoring exercise projects to oblige these limits is fundamental for further developing adherence [26].

Despite motivation, certain factors prevent adherence. Time constraints and fatigue are very prominent factors, particularly among multiple comorbidity participants [27]. Psychological barriers (e.g. boredom, lack of interest, and social stigma), especially among women, discourage exercise adherence [28]. 

Limitations and future research

While this study gives important insight, it isn't without constraints. The cross-sectional study design collects data at a single point in time, which limits its ability to establish cause-and-effect relationships between identified factors and adherence over time. Convenience sampling may limit representativeness. Furthermore, the reliance on self-reported data may introduce certain biases, such as social desirability bias or recall bias, as participants might underreport or overreport their activity levels. Future research could profit from a longitudinal study to follow changes in practice adherence over the long term.

Further investigations are needed to understand specific social and natural factors that impact practice adherence in Saudi Arabia. Qualitative investigations could give further insight into the individual and social dynamics influencing exercise and assist in developing adherence.

Future research and recommendations

Longitudinal studies are recommended to evaluate the long-term effects of exercise on glycemic control and overall health outcomes, giving a more complete comprehension of the advantages of physical activity. Based on our study, we hypothesize that assessing interventional procedures, for example, tailoring exercise programs to specific needs and supportive groups, can assist with further development in exercise adherence. Exploring how social and financial factors impact exercise adherence can prompt more comprehensive and socially delicate projects. Contrasting exercise adherence and results between various populations or regions can help identify the best practices for physical activity.

## Conclusions

This study highlights a low level of exercise adherence among diabetic patients in Saudi Arabia. Walking was the most preferred form of physical activity, due to its accessibility and ease of integration into daily life. Participants of younger age, men, and individuals with better glycemic control demonstrated high adherence to exercise. Key facilitators included social support and access to resources, while major barriers involved time constraints and health-related limitations. Addressing these challenges can play a crucial role in improving exercise adherence and, consequently, diabetes management outcomes.

## Appendices

Questionnaire 

First Section

Gender 

- Male 

- Female 

Age 

- write in numbers only

Nationality

-Saudi 

-Non Saudi

Marital  status 

Single

Married 

Divorced 

Widow /widower 

Region 

Middle 

Eastern

Western 

Northern 

Southern

-----------------

Second Section

What Type of diabetes mellitus do you have?

Type 1

Type 2

For how long did you have it?

Less than 1 year

1-3 years

3-5 years

5-10 years

More than 10 years

What type of medication/therapy are you on?

Lifestyle modifications(exercise and healthy die)

Pills that lower sugar level

Pills that lower sugar level and insulin injections

Insulin injections

Is there a family history of diabetes?

Yes

No

Blood glucose level(HbA1c) 

Less than 7%

7 - 8.5%

8.5 - 10%

More than 10%

Did you receive educational awareness about diabetes from a specialized doctor?

Yes

No

Did the doctor recommend to you to perform exercise for about 150 mins in a week?

Yes

No

What type of exercise did the doctor recommend for you to perform?

Running/jogging

Bicycle riding 

Swimming

Competitive sports (football, basketball…etc)

Weight lifting 

Other:

Did you exercise before you were diagnosed with diabetes?

Yes 

No 

Do you measure the blood glucose level before exercise?

Always

Most of the time

Sometimes 

Rarely 

Never

If you find the blood glucose level less than 90, what do you do?

I stop exercising 

I start exercising right away

I consume sugars before exercising (candy, biscuit, or juice)

I stop consuming any sugars before exercising

Other:

If you find the blood glucose level between 90-250, what do you do?

I stop exercising 

I start exercising right away

I consume sugars before exercising (candy, biscuit, or juice)

I stop consuming any sugars before exercising

Other:

If you find the blood glucose level more than 250, what do you do?

I stop exercising 

I start exercising right away

I consume sugars before exercising (candy, biscuit or juice)

I stop consuming any sugars before exercising

Other:

-----------

Third Section

Do you perform any of the following exercises

Rope jumping

Walking 

Jogging/running 

Bicycle riding

Other:

How many times a week do you perform aerobic exercise?

5-6 times a week

3-4 times a week

1-2 times a week

I don’t perform it

How much time do you spend doing it?

Less than 30 mins

30 mins to 1 hour

1-2 hours

More than 2 hours

I don’t perform it

Do you perform resistance exercises?

Yes

No 

I don’t know any exercises 

What type of resistance exercise?

Using my Body weight 

Resistance bands

Weight lifting

I don’t perform it

How many times a week do you perform resistance exercise?

5-6 times a week

3-4 times a week

1-2 times a week

I don’t perform it

How much time do you spend doing it?

Less than 30 mins

30 mins to 1 hour

1-2 hours

More than 2 hours

I don’t perform it

-----------------------

Fourth Section

What are the factors that encourage you to perform this type of exercise?

Can be done at home

Less money is spent

Lower risk of injury

Not exhausting 

Results of this exercise are effective in controlling blood glucose and lowering body weight  

Other:

What are the factors that discourage you from performing this type of exercise?

Difficult to perform

Expensive

Fear of injury

Exhausting 

Results of this exercise are not effective in controlling blood glucose and lowering body weight  

Other:

What are the factors that stop you from exercising?

I think medications are enough to treat my diabetes 

My health status prevents me from exercising

I am worried that I might get injured 

No time to exercise

The weather prevents me from exercising

Gym memberships are expensive

Other:

Did you have any support from your community to exercise?

Yes

No 

If you chose no, what are the reasons?

Too many social events

No one from my community exercises

Exercise is not appropriate for women

What are the factors that encourage you to keep exercising?

The educational campaigns about exercising 

I am in a supportive environment (I have a partner to exercise with, moral support, different types of exercise)

Exercise is one of the top priorities in my life

Having a role model

Part of my goals (losing weight, improving fitness, improving quality of life)

Other:
